# *Toxoplasma gondii* in Sheep and Goats from Central Iran

**DOI:** 10.1186/s13104-021-05465-3

**Published:** 2021-02-04

**Authors:** Mojtaba Bahreh, Bahador Hajimohammadi, Gilda Eslami

**Affiliations:** 1 Research Center for Food Hygiene and Safety, School of Public Health, Shahid Sadoughi University of Medical Sciences, Yazd, Iran; 2 International Campus, Department of Food Hygiene and Safety, School of Public Health, Shahid Sadoughi University of Medical Sciences, Yazd, Iran; 3 Department of Food Hygiene and Safety, School of Public Health, Shahid Sadoughi University of Medical Sciences, Yazd, Iran; 4 Department of Parasitology and Mycology, School of Medicine, Shahid Sadoughi University of Medical Sciences, Yazd, Iran

**Keywords:** Toxoplasmosis, Foodborne disease, Nested PCR, *Toxoplasma gondii*

## Abstract

**Objective:**

Toxoplasmosis, caused by *Toxoplasma gondii*, infects humans by consuming infected raw or undercooked meat and foods harboring mature oocysts. In this study, we assessed the prevalence of *T. gondii* in sheep and goats coming from central Iran. After completing the questionnaire, about one gram of liver or diaphragm tissue was taken as a sample from 90 sheep and 90 goats slaughtered in Yazd Province and stored at – 20 ºC. DNA extraction was done, and then *T. gondii* was detected using nested PCR.

**Results:**

This study indicated that the prevalence of *T. gondii* in all slaughtered animals was 11.6% (21 of 180), including 14.4% (13/90) in sheep and 8.8% (8/90) in goats. The infection rates in liver and diaphragm samples were 12.2% (11/90) and 11.1% (10/90), respectively (*p* = 0.8163). The infection rate in animals older than one was 16.3% (15/92), and it was 6.8% (6/88) in animals under one year of age. Therefore, no significant differences were found (*p* = 0.475). Infection rates were 19.5% (18/92) in males and 3.4% (3/88) in females (*p* = 0.0007). In conclusion, the infection rates of toxoplasmosis in livestock in this area are almost high, and therefore, it is necessary to design appropriate prevention programs to control the disease.

## Introduction

Toxoplasmosis, as a widespread zoonotic disease worldwide, is caused by *Toxoplasma gondii* [1]*.* This protozoan belongs to the phylum Apicomplexa, which infects at least one-third of the human population [2]. *T. gondii* has a wide range of host species [3]. Besides, toxoplasmosis is considered the third disease globally, resulting in human hospitalization due to foodborne infection. In Iran, the mean seroprevalence rate of toxoplasmosis is 39.3% in the human population [4].

*T. gondii* life cycle includes three infectious stages; oocyst harboring sporozoite, shedding from the cats, and felids’ feces as the definitive host, tachyzoite an active form in toxoplasmosis, tissue cyst inside the muscle and central nervous system [3]. This foodborne parasite may infect humans by consuming food and water contaminated by mature oocyst or eating rare or semi-rare cooked meat, or products with tissue cysts.

Meat coming from domestic animals harboring *T. gondii* is considered an important infection source in humans [5]. Pregnant women and anemic patients show interest in consuming raw meat and its products, liver, and other organs as they believe in their health benefits.

The prevalence of *T. gondii* in sheep and goats in different areas is between 24.5 and 33.3% [6–8]. Overall, the prevalence of toxoplasmosis is reported in the range of 28.5–78% among ovine worldwide [9, 10].

*T. gondii* is detected using fluorescent microscopy, serology tests, and molecular-based methods. The fluorescent microscopy is accurate, but it needs the live parasite. Besides, the serology tests are sensitive, but they yield false-negative results in some cases. Consequently, molecular-based techniques are considered the most sensitive and specific methods [5] and were chosen as accurate and suitable methods for detecting this disease.

*T. gondii* was identified in goats and sheep using the PCR-based method, which may help assess the consumer’s infection risk. Hence, in the present study, the prevalence of *T. gondii* in sheep’s and goats’ meat slaughtered in the slaughterhouse of central Iran was evaluated using the PCR-based method. Based on our knowledge, this is the first report of molecular detection of *T. gondii* in goats and sheep tissue using the nested-PCR assay in Central Iran.

## Main text

### Methods

#### Area description

The samples were obtained from the slaughterhouses in Yazd, the Yazd Province capital, located in central Iran. Its climate is dry and hot, with an average annual rainfall of 60 mm, and an average humidity percentage of 31.5%. Yazd is placed at 1203 m above sea-level and covers 16,000 km^2^.

#### Sampling

The samples were collected from the slaughterhouses of Yazd city. For this purpose, one g of the liver or diaphragm tissue of 90 slaughtered goats and 90 slaughtered sheep was taken as the sample. All samples were stored at − 20 ºC until the next step. Data such as age, gender, and location were recorded in the questionnaire.

#### DNA extraction

The salting-out method was used based on the protocol introduced in previous studies to extract genomic DNA [11, 12] with some modifications. Briefly, about 30 mg of the sample was used for DNA extraction. NET buffer (Tris–HCl, pH 7.6, 50 mM; EDTA, pH 8, 25 mM; NaCl, 50 mM) was used for complete lysis of the tissue. About 900 μl NET buffer plus 10 μl proteinase K (20 mg/ml) were added to each tube. After the vortex, each tube was incubated at 56 °C overnight. After centrifugation, the supernatant was transferred to a new sterile 1.5 ml tube. For purification, 300 μl saturated NaCl was added and mixed for 30 s. Centrifugation was done for 10 min at 10,000 rpm, and then the supernatant was transferred to a new sterile 1.5 ml tube. For DNA precipitation, two volumes of cold ethanol were added to each sample. After vortex, each sample was stored at − 20 °C for at least 30 min. The centrifugation was done at 15,000 rpm for 10 min, and the supernatant was removed entirely. Afterward, the washing step was done with 70% cold ethanol. It should be mentioned that the extracted genomic DNA was soluble in TE buffer (Tris–HCl, pH 7.6, 10 mM; EDTA, pH 8, 1 mM). For quality and quantity evaluation of the extracted DNA, the agarose gel electrophoresis (0.8%; Akhtarian, Tehran, Iran) and spectrophotometer (NanoDrop; Thermo Scientific, USA) were used, respectively. The extracted DNA was stored at − 20 °C for the next experiments.

#### Nested PCR

To detect *T. gondii*, the nested PCR was performed using the specific primer pairs B1: 5′-GGAACTGCATCCGTTCATGAG-3′ and B2: 5′-TCTTTAAAGCGTTCGTGGTC-3′ for the first step and B3: 5′-TGCATAGGTTGCAGTCACTG-3′ and B4: 5′-GGCGACCAATCTGCGAATACACC-3′ for the second round [13]. Amplification reaction for the first round was performed in a volume of 20 μl containing 1 × master mix PCR (Ampliqon, Odense, Denmark), 0.5 μM each primer (Pishgam, Tehran, Iran), and 100 ng extracted DNA. The second round’ amplification reaction was the same as the first round, but 1 μl PCR product as the template. The amplification program for both rounds was 94 °C for 5 min as the first denaturation, then followed by 35 cycles of denaturation at 94 °C for 1 min, annealing at 55 °C for 1 min, elongation at 72 °C for 1 min. The final extension was done at 72 °C for 5 min. The amplification analysis was done using 3% agarose gel electrophoresis. The fragment size of 94 bp in length showed *T. gondii*. For all rounds, positive and negative controls were used. The standard strain of *T. gondii* was purchased from Pasteur Institute, Tehran, Iran. The ddH_2_O was used for negative control.

### Statistical analysis

Statistical analysis was performed using SPSS version 16.0, and the *χ*^2^ test was used to analyze the relationship between each variable, including sex, age, and parasite position with the toxoplasmosis infection. The significance level was considered as *p* < 0.05.

## Results

**Fig. 1:**
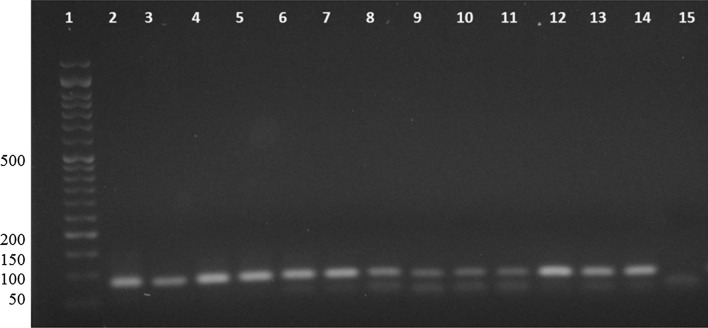
Agarose gel electrophoresis for analysis of nested PCR for *Toxoplasma gondii* identification. The amplicon size 94 bp in length denotes *T. gondii*. Lane 1: 50 bp DNA ladder; lane 2: positive control (standard *T. gondii* from Pasteur Institute, Tehran, Iran); lanes 3–14: the samples with *T. gondii*; lane 15: negative control (ddH_2_O)

The nested PCR showed an amplicon of 94 bp in length in 21 samples (Fig. 1). Therefore, the prevalence of *T. gondii* in all slaughtered animals was 11.6% (21 of 180), including 14.4% (13 of 90) in sheep and 8.8% (8 of 90) in goats. The infection rate by *T. gondii* in liver and diaphragm samples were 12.2% (11 of 90) and 11.1% (10 of 90), respectively, which is not significant (*p* = 0.8163; Table 1). The analysis revealed that 16.3% (15 of 92) of the infected slaughtered animals were more than one year old, and 6.8% (6 of 88) were under one year of age. There were no significant differences between infection rate and age (*p* = 0.475; Table 1). The sex analysis showed that 19.5% (18 of 92) of males and 3.4% (3 of 88) of females were infected with *T. gondii* (*p* = 0.0007; Table 1).

**Table 1:** The relationship between infection with *Toxoplasma gondii* and kind of animals, sex, age, and tissue type

	Animal			Sex			Age			Tissue		
	Sheep	Goats	*p* value	Male	Female	*p* value	> 1	< 1	*p* value	Liver	Diaphragm	*p* value
	No. (%)	No. (%)		No. (%)	No. (%)		No. (%)	No. (%)		No. (%)	No. (%)	
*T. gondii*	13 (14.4)	8 (8.9)	0.25	18 (20.4)	3 (3.2)	0.0007	15 (16.3)	6 (6.8)	0.475	11 (12.2)	10 (11.1)	0.8163
Total	21 (11.7)			21 (11.7)			21 (11.7)			21 (11.7)		

## Discussion

Meat is one of the most important sources of animal protein in the human diet. However, it may be infected or contaminated by various types of pathogens, including bacteria, prions, fungi, and parasites, which may infect human consumers and lead to hazardous health problems. The parasitic infection in livestock has unsuitable effects on the meat industry’s quality and threatens human health [13]. Several studies showed the prevalence of *T. gondii* in different kinds of meat [14–19]. In this regard, Boughattas et al. [20] studied the prevalence of *T. gondii* in humans who consumed sheep meat in Tunis using serological and PCR methods. Using the serological method, they showed that 38.2% of young sheep and 73.6% of the adult ones are infected, and using the PCR-based method, they found out that 50% of the samples from sheep tissues are HIV-positive [20]. Gebremedhin et al. [21] studied the prevalence of *T. gondii* infection in sheep and goats slaughtered in Ethiopia using the direct agglutination test (DAT), in which the prevalence of the parasite was reported 17.68% (111/628). They showed that the risk of infection with *T. gondii* in younger sheep is significantly higher than in adult ones, and the infection was higher during the wet seasons [21]. Our study results also indicated infection in adult sheep and the lambs, but there was no significant difference between infection rate and age. Khezri et al. [22] assessed the prevalence rate of *T. gondii* in sheep in different Kurdistan regions using a serological test, such as indirect ELISA. They reported that out of 368 sheep, 21.74% are serologically positive, among which 20.87% were male, and 22.13% were female sheep. Their analysis showed no significant difference between the sex and the rate of infection. However, we found a significant difference between age and the infection rate. Asgari et al. [23] identified the prevalence rate of infection in the edible tissues of the brain, tongue, liver, neck, ribs, and femur from 56 sheep and 22 goats in Shiraz using the PCR with an infection rate of 37.5% (21/56) and 22.7% (5/22), respectively. The overall prevalence of infection in animals was estimated to be 33.3%. This infection rate was almost the same as the infection rate in our study. The rate of infection in females and males was 46% (9 of 14) and 29.5% (12 of 45), respectively, which is significantly different (*p* = 0.013). This study showed a significantly higher infection rate in males. One possible cause of this significant difference would be the difference in female care locations to provide milk. Therefore, the risk of contamination with oocysts expelled from cats will be decreased. Asgari et al. [23] also showed that the highest infected tissue in an orderly manner is the tongue (21.8%), brain (20.19%), and femoral intercostal muscles (9.17%). In the current study, there was no significant difference between location and the infection. The findings showed that 12.2% of infection is in the liver; however, the consumption of rare or semi-rare sheep liver is traditionally common in the studied area. Thus, it is necessary to inform pregnant women and immune-compromised people to be careful while consuming rare or semi-rare sheep offal and liver. Seemingly, the essential way to prevent infection is educating people at higher risk about the life cycle of this parasite, informing people about consuming raw or undercooked meat, liver, and offal, preserving the meat either at − 20 °C for 24 h or at − 4 °C for 48 h before consumption, and cooking meat and its products at 70 °C.

## Limitation

This study investigated the presence of *T. gondii* in goats’ and sheep’s meat. It is essential to design a program to prevent this infection in the region. This study’s limitation was the small sample population used to analyze the accurate relationship between the infection rate and age, sex, and livestock parasite position.

## Data Availability

The datasets used and/or analyzed during the current study available from the corresponding author on reasonable request.

## Abbreviations

*T. gondii*: *Toxoplasma gondii*
PCR: Polymerase Chain Reaction
NET: NaCl, EDTA, Tris–HCl
